# Development and evaluation of an EHR‐based computable phenotype for identification of pediatric Crohn's disease patients in a National Pediatric Learning Health System

**DOI:** 10.1002/lrh2.10243

**Published:** 2020-08-28

**Authors:** Ritu Khare, Michael D. Kappelman, Charles Samson, Jennifer Pyrzanowski, Rahul A. Darwar, Christopher B. Forrest, Charles C. Bailey, Peter Margolis, Amanda Dempsey, And the PEDSnet Computable Phenotype Working Group

**Affiliations:** ^1^ IQVIA Plymouth Meeting Pennsylvania USA; ^2^ Division of Pediatric Gastroenterology, Department of Pediatrics University of North Carolina at Chapel Hill Chapel Hill North Carolina USA; ^3^ Division of Gastroenterology, Hepatology & Nutrition; Department of Pediatrics Washington University in St Louis School of Medicine St. Louis Missouri USA; ^4^ Adult and Child Consortium for Outcomes Research and Dissemination Science University of Colorado Denver Aurora Colorado USA; ^5^ Applied Clinical Research Center Children's Hospital of Philadelphia Philadelphia Pennsylvania USA; ^6^ Applied Clinical Research Center, Children's Hospital of Philadelphia, Philadelphia PA and Department of Pediatrics, Perelman School of Medicine University of Pennsylvania Philadelphia Pennsylvania USA; ^7^ James M. Anderson Center for Health Systems Excellence, Department of Pediatrics Cincinnati Children's Hospital Medical Center Cincinnati Ohio USA; ^8^ Department of Pediatrics University of Colorado Denver Aurora Colorado USA

**Keywords:** computable phenotype, Crohn's disease, electronic health records, PEDSnet

## Abstract

**Objectives:**

To develop and evaluate the classification accuracy of a computable phenotype for pediatric Crohn's disease using electronic health record data from PEDSnet, a large, multi‐institutional research network and Learning Health System.

**Study Design:**

Using clinician and informatician input, algorithms were developed using combinations of diagnostic and medication data drawn from the PEDSnet clinical dataset which is comprised of 5.6 million children from eight U.S. academic children's health systems. Six test algorithms (four cases, two non‐cases) that combined use of specific medications for Crohn's disease plus the presence of Crohn's diagnosis were initially tested against the entire PEDSnet dataset. From these, three were selected for performance assessment using manual chart review (primary case algorithm, n = 360, primary non‐case algorithm, n = 360, and alternative case algorithm, n = 80). Non‐cases were patients having gastrointestinal diagnoses other than inflammatory bowel disease. Sensitivity, specificity, and positive predictive value (PPV) were assessed for the primary case and primary non‐case algorithms.

**Results:**

Of the six algorithms tested, the least restrictive algorithm requiring just ≥1 Crohn's diagnosis code yielded 11 950 cases across PEDSnet (prevalence 21/10 000). The most restrictive algorithm requiring ≥3 Crohn's disease diagnoses plus at least one medication yielded 7868 patients (prevalence 14/10 000). The most restrictive algorithm had the highest PPV (95%) and high sensitivity (91%) and specificity (94%). False positives were due primarily to a diagnosis reversal (from Crohn's disease to ulcerative colitis) or having a diagnosis of “indeterminate colitis.” False negatives were rare.

**Conclusions:**

Using diagnosis codes and medications available from PEDSnet, we developed a computable phenotype for pediatric Crohn's disease that had high specificity, sensitivity and predictive value. This process will be of use for developing computable phenotypes for other pediatric diseases, to facilitate cohort identification for retrospective and prospective studies, and to optimize clinical care through the PEDSnet Learning Health System.

**Abbreviations**
CP: computable phenotype
EHR: electronic health record
ICD9/10: International Classification of Disease 9/10
OMOP: observational medical outcomes partnership
SNOMED‐CT: systematized nomenclature of medicine—clinical terms

## 1 INTRODUCTION

The era of using “Big Data” for research has resulted in many new opportunities to harness the power of electronic medical records and other complex data sets for improving child health. This is especially important because, compared to adults, there is a paucity of large, high quality data sources available for research on pediatric issues. Developed in 2014, PEDSnet (PEDSnet.org) is a multi‐institutional Learning Health System that aggregates electronic health record (EHR) data from eight of the nation's largest children's health systems,^1^ and currently comprises over six million U.S. children. PEDSnet data is transformed into a common data model that allows for direct comparison of clinical records across health systems. Because of this, and because PEDSnet undergoes rigorous quality checks and is a relatively comprehensive data source describing patients' clinical care, it holds great promise as a valuable child health research tool for defining disease cohorts.

The ability to accurately identify patients with specific medical conditions is essential for efficiently constructing study cohorts needed for retrospective or prospective observational studies, and for implementing evidence‐based interventions in Learning Health Systems. In addition, screening phenotypes can also be used across large populations to aid in recruiting patients for prospective clinical trials. However, for large multi‐center populations, manually gathering this data across different data platforms and health systems becomes difficult or infeasible. In order to efficiently examine a large amount of data from many clinical systems and institutions, it becomes necessary to express eligibility criteria as an algorithm that can be automated. This process, called “computable phenotyping,” uses multiple EHR data elements such as laboratory results, medications, procedures, biometrics, clinician notes, and vital signs to define a cohort of interest.^2^

Because PEDSnet and similar collaborative networks use a common data model to achieve semantic interoperability across systems, effective computable phenotypes have the potential for greater standardization and reuse than site‐specific cohort ascertainment. In this study, Crohn's disease was chosen as a “use case” for creation of a computable phenotype because it is a childhood disease with several potential therapeutic treatment options, but with limited data on comparative effectiveness from clinical trials and, few previously defined computable phenotype algorithms. Moreover, because Crohn's disease is a relatively rare condition, finding adequate numbers of patients through traditional methods as potential participants for such trials can be time consuming and expensive.^3^ Developing a computable phenotype for Crohn's disease that can effectively and efficiently detect cohorts with this disease for future clinical trials, and dissemination of evidence‐based interventions is therefore a high research priority.^4^, ^5^

## 2 RESEARCH FOCUS

The purpose of this study was twofold: (a) to describe a process for using EHR data to develop and evaluate discrete‐data computable phenotypes that could potentially be applied to other diseases and conditions; and (b) to develop and validate a computable phenotype specifically for Crohn's Disease to support case‐finding for future research.

## 3 METHODS

The Children's Hospital of Philadelphia Institutional Review Board approved all study activities (approved protocol 16‐012878) and the other PEDSnet institutions relied on that determination. All analyses were completed using R 3.3.1 and Postgres 9.5.

### 3.1 Dataset

The PEDSnet^1^ data network includes structured EHR data for patients who have had at least one face‐to‐face encounter and at least one physician‐recorded diagnosis in or after 2009. Data are transformed into the OMOP common data model,^6^ a widely used data model for representing secondary datasets drawn from electronic health records, which uses standard terminologies (eg, SNOMED CT for diagnoses, and RxNorm for medications) as well as data structures. In PEDSnet, the multi‐site aggregated dataset is built iteratively every 3 months via a data coordinating center, which conducts data quality assessments^7^, ^8^, ^9^ on each release, and provides feedback to the sites for improving the individual datasets for the next iteration. Data for this study came from the May 2016 and March 2017 releases of PEDSnet, both of which included patients' retrospective data back to 2009.

### 3.2 Computable phenotype development process overview

Development of the Crohn's disease computable phenotype was done by defining a target case (ie, a patient with Crohn's disease) and non‐cases that were lacking some or all the target criteria but were similar in other ways to cases, in order to optimize the algorithm's discriminant ability. For this work, we examined two non‐case algorithms—(a) patients with a gastrointestinal diagnosis other than Crohn's disease, ulcerative colitis or indeterminate colitis seen by any specialty or (b) patients with a diagnosis other than Crohn's disease, ulcerative colitis, or indeterminate colitis seen specifically by a gastroenterologist/in a gastroenterology clinic.

Features to be tested in the various Crohn's disease algorithms were selected using disease expert and informatician input, with the goal of investigating the value of different data types. In order to evaluate different selection criteria, initially six alternative versions of the case algorithm were iteratively developed and assessed by cross referencing against a highly vetted, validated, national disease registry using data from one participating PEDSnet institution. This registry, supplied by the Improve Care Now network is considered a highly reliable source for identifying Crohn's disease patients as it represents a list of patients with a definitive diagnosis of Crohn's disease as determined by gastroenterologists, and in which the data undergoes rigorous external validation. Presence on this list was considered as an initial “gold standard” for selecting case algorithms to assess further, and this selection was based on computed positive predictive value (PPV) and sensitivity. The algorithms, ranging from one diagnosis (1d) through six diagnoses with medications (6 dm), identified the version that delivered the optimal tradeoff between sensitivity and specificity, rather than optimizing for one performance characteristic. This criterion was chosen because seeking a computable phenotype with this balance would permit the phenotype to be used for both case screening, without a high risk of missing true positives, and case selection, without a high risk of including false positives.

Based on these results, four case algorithms were selected for further validity testing that included an assessment of the demographic distributions among the populations captured, and logistic regression models assessing the odds of a patient having an endoscopy or other radiographic exams focused on the gastrointestinal system (ie, upper GI series). Based on the results from this further testing, one primary algorithm and one alternative algorithm for cases and one primary algorithm for non‐cases were chosen and externally validated across all sites using chart reviews. The small number of chart reviews for the alternative case algorithm was undertaken in order to understand discriminant validity between two closely related Crohn's disease algorithms.

### 3.3 Exposure selection

#### 3.3.1 Diagnosis terms

Since Crohn's disease is a chronic condition with specific diagnostic codes and therapy, the starting criterion for case algorithms was physician‐recorded diagnosis. We used as a seed for assembling codesets the ICD‐9‐CM codes described by Ritchie et al.^10^ for Crohn's disease (555.x; 4 codes), ulcerative colitis (556.x; 9 codes), and gastrointestinal disease (008.x, 009.x, 578.x, 579.x, 787.x, 564.x, 531.x, 532.x, 533.x, 534.x, 535.x, 536.x, 530.x, 564.x, 579.x; 253 codes). The seed codes were used to identify the SNOMED‐CT analogs using the UMLS‐based OMOP vocabulary crosswalk. In addition, keyword searches (eg, include “Crohn” and exclude “non‐Crohn” and “remission”) identified additional SNOMED‐CT terms. Finally, the SNOMED‐CT hierarchy was manually reviewed to identify relevant ancestors and descendants of the terms identified. The final diagnosis codesets included 49 terms for Crohn's disease, 34 terms for ulcerative colitis, and 8280 terms for other gastrointestinal diagnoses (available in Supplemental Material for Crohn's and ulcerative colitis, and from the authors for other gastrointestinal diagnoses.)

#### 3.3.2 Medications

The presence or absence of medications often used in Crohn's disease was used as a potential exposure for defining cases and non‐cases. Medications included^10^ balsalazide, mesalamine, sulfasalazine, ciprofloxacin, levofloxacin, metronidazole, rifaximin, prednisone, budesonide, azathioprine, mercaptopurine, methotrexate, infliximab, adalimumab, certolizumab, and natalizumab. To define the medication codeset, we began with RxNorm ingredient‐level codes, then all clinical drug forms were derived and limited to relevant routes of administration for a Crohn's disease indication, viz. oral, intravenous, intramuscular, subcutaneous, or rectal. Finally, the drug concepts, including descendants, were derived from the RxNorm taxonomy (https://www.nlm.nih.gov/research/umls/rxnorm/) using standard methods.^11^

### 3.4 Test algorithm design

Six different Crohn's disease case algorithms were initially compared against a Crohn's disease registry at a single institution to begin to understand the interaction between sensitivity, specificity and number of times a diagnosis or medication was encountered in the chart (data not shown). From this we narrowed the potential algorithms down to four case and two non‐case algorithms, which used a combination of diagnoses and medications. Data from these four algorithms are the focus of the manuscript. All case algorithms required a Crohn's disease diagnosis recorded during a physician visit or specified as a problem list entry at least one or more times in the dataset, representing different encounters. Diagnoses captured during other, non‐face‐to‐face or non‐physician encounter types (eg, telephone encounters, lab‐ or x‐ray‐ only visits) were excluded to reduce the risk of inaccurate or unreliable diagnoses. Some of the test algorithms also included use of any Crohn's disease medications (at any point in time). Table 1 illustrates the use of different combinations of exposures to design different versions of the case (n = 4 algorithms) and non‐case algorithms (n = 2).

**TABLE 1:** Test algorithms for Crohn's disease computable phenotyping

	Exposures				
Algorithm	Diagnosis	Diagnosis encounter type	Specialty care	Medication	Exclusion diagnosis
1+ Diagnosis codes	Crohn's disease	One or more in‐person encounters or problem list entry	‐	‐	Number of ulcerative colitis encounters > Number of Crohn's disease encounters
1+ diagnosis codes and 1+ medication codes	Crohn's disease	One or more in‐person encounters or problem list entry	‐	Crohn's disease medications	Number of ulcerative colitis encounter > Number of Crohn's disease encounters
3+ diagnosis codes	Crohn's disease	Three or more in‐person encounters or problem list entries	‐	‐	Number of ulcerative colitis encounters > Number of Crohn's disease encounters
3+ diagnosis codes and 1+ medication code	Crohn's disease	Three or more in‐person encounters or problem list entries	‐	Crohn's disease medications	Number of ulcerative colitis encounters > Number of Crohn's disease encounters
Non‐case from general population	GI disease	One or more in‐person encounters	‐	‐	Any diagnoses of Crohn's disease, ulcerative colitis, indeterminate colitis
Non‐case selected from patients seen by a pediatric gastroenterologist	GI disease	One or more in‐person encounter	Encounter with a GI provider or at a GI care site	‐	Any diagnoses Crohn's disease, ulcerative colitis, indeterminate colitis

Because the distinction between Crohn's disease and ulcerative colitis can be difficult to determine, and it is not uncommon for a patient to have both diagnoses recorded at some point in their chart,^12^ we excluded patients who had a greater number of encounters with a diagnosis of ulcerative colitis than Crohn's disease to minimize false positives.

**FIGURE 1:**
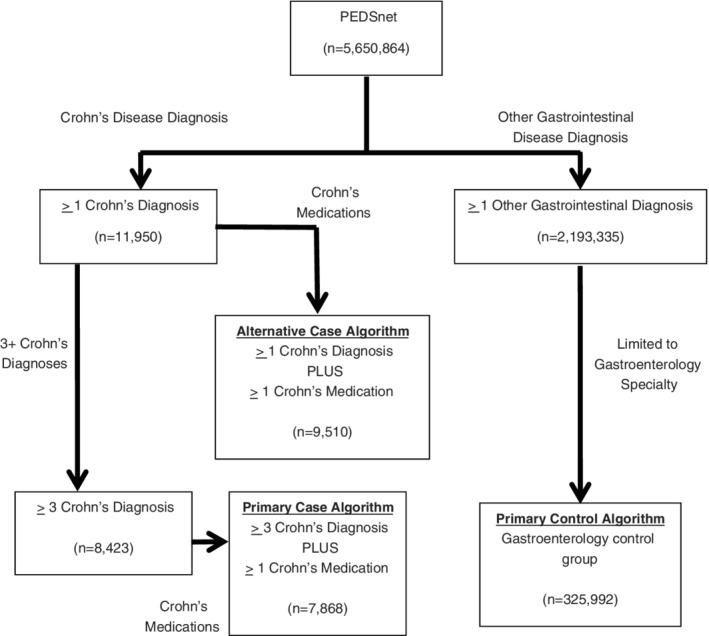
PEDSnet population distribution across Crohn's disease computable phenotype case and non‐case algorithms

Non‐case test algorithms included patients with a gastrointestinal diagnosis other than Crohn's disease, ulcerative colitis, or indeterminate colitis seen at an in‐person encounter. One non‐case test algorithm included patients seen by any medical specialty (general population) whereas the other (called “non‐case gastroenterology”) required having a visit specifically with a gastroenterologist or in a gastroenterology specialty care site (Figure 1). Specialty was determined as the specialty of the physician recording the diagnosis, or the specialty of the location of the encounter if physician specialty was not recorded. In the final analysis, non‐cases were defined by the “non‐case gastroenterology” algorithm to help assess the discriminant validity of the case algorithm from “near cases”—that is patients with similar clinical features and care settings as cases but with different final diagnoses.

### 3.5 Reliability and validity of test algorithms

We conducted reliability testing across years to assess the stability of the computable phenotype by examining the proportion of cases that had at least one additional follow‐up visit for Crohn's in any subsequent year. To examine known‐group validity, patient age distributions and sex ratios were assessed to determine if any of the Crohn's disease algorithms generated cohorts with unexpected distributions of these variables.^13^ In addition, logistic regression was used to test hypotheses regarding associations between the count of Crohn's disease encounters (1, 2, 3, 4, 5+ occurrences) plus any use of Crohn's medications (infliximab and adalimumab), gastrointestinal endoscopy, and gastrointestinal fluoroscopic exam. Non‐case comparators in these regression analyses were derived from the “non‐case gastroenterology” algorithm.

### 3.6 Chart review

At each PEDSnet institution, we conducted manual chart reviews of a random selection of 45 case patients using the final algorithm (3+ diagnosis codes plus 1+ medications) and 45 non‐case patients from the gastroenterology clinic population. The charts of an additional randomly selected 10 patients from each institution with an alternative case definition algorithm (1+ Crohn's disease diagnoses and 1+ medications) were also reviewed. Institutional chart reviewers received identical training and used an evaluation algorithm that systematically assessed different components of the electronic medical record (ie, problem list, past diagnoses, medications, chart notes) to determine whether the patient had a definitive diagnosis of Crohn's at the most recent gastroenterology visit (some patients had an evolution of their symptoms such that they were initially diagnosed with indeterminate colitis or ulcerative colitis before a definitive diagnosis of Crohn's disease was made by the clinician). Chart review data were entered directly into a Research Electronic Data Capture (REDCap)^14^ database hosted at the Children's Hospital of Philadelphia. Patients were determined as “true cases” based on pre‐specified criteria developed by the study team using data elements collected by the chart reviewers. Research assistants conducted the initial chart reviews, and were blinded as to whether the patient was categorized as a case or non‐case. When the diagnosis of Crohn's were not clear from these data, clinicians from the study team reviewed all data in the chart to make a final determination about the diagnosis of Crohn's. In addition, all false positives and false negatives were further examined by the same two clinicians from the study team to identify patterns of error. The sensitivity, specificity, and positive predictive values (PPV) were computed for the primary case and primary control algorithms as per the standard definitions.

## 4 RESULTS

Figure 1 illustrates the PEDSnet population and the distribution across selected versions of the case and non‐case algorithms. There were 11 950 patients with at least one diagnosis of Crohn's disease. The addition of a medication constraint to a single diagnosis reduced the cohort size by 20%; when >1 diagnosis was required, the impact of adding a medication constraint decreased. There were over two million non‐cases seen among any medical specialty, and 325 992 seen specifically in gastroenterology.

### 4.1 Comparison to a disease registry

As expected, with comparison to a Crohn's disease registry from one of the PEDSnet sites the least specific algorithm version led to highest sensitivity, and the most specific algorithm version led to the highest precision (Figure S1). The false negatives (those who were on the list but not identified by the algorithm) represented early onset patients or the patients who did not follow‐up after 2009, the cut‐off date in PEDSnet inclusion criteria. The false positives (those identified by the algorithm but not on the list) existed partly due to the latency of data capture in the list, and partly due to one patient's disease going into sustained remission (ie, had a stem cell transplant). The addition of medications to the algorithm tended to eliminate patients with multiple visits where symptoms potentially consistent with Crohn's were being evaluated but in whom a different diagnosis was ultimately found. Based on these results, the 3+ diagnosis codes plus 1+ medications algorithm was selected as the primary case algorithm for further testing as it appeared to have the best balance between precision and sensitivity, with 1+ diagnosis codes plus 1+ medications  assessed as an alternative case algorithm that would maximize sensitivity.

### 4.2 Validity and reliability

Table 2 describes results of the initial validity assessment of the four cohorts resulting from the four different computable phenotype algorithms assessed for cases. Adolescent age, slight male predominance, and use of TNFα antagonists were consistent with expectation and varied across the different algorithms assessed.^13^ The proportion of Crohn's disease patients with follow up visits, an indirect measure of case identification reliability, ranged from 76% to 87% (Table 2). The presence of Crohn's disease diagnosis codes and medications were significantly associated with both endoscopy and GI related radiographs (Table S1).

**TABLE 2:** Crohn's disease phenotype population description across the four algorithms tested (average across sites, with ranges)

	Computable phenotype			
	1+ diagnosis N = 11 950	1+ diagnosis and 1+ medications N = 9510	3+ diagnoses N = 8423	3+ diagnoses and 1+ medication N = 7868
Prevalence per 10 000	21.17 (9.92‐36.4)	16.62 (7.77‐25.54)	14.91 (6.9‐19.96)	13.83 (6.31‐20.67)
Current age (years)	19.15 (18.13‐20.6)	19.21 (18.61‐20.74)	19.32 (18.29‐21.14)	19.37 (18.17‐21.14)
Sex ratio (male/female)	1.28 (1.04‐1.33)	1.32 (1.11‐1.45)	1.36 (1.27‐1.47)	1.38 (1.2‐1.56)
Infliximab prevalence (%)	20.97 (1.14‐31.64)	26.34 (1.56‐42.02)	27.98 (1.43‐44.94)	29.81 (1.63‐48.99)
Adalimumab prevalence (%)	16.62 (4.98‐24.02)	19.83 (6.36‐30.47)	21.73 (6.56‐33.63)	23.34 (7.17‐34.69)
Crohn's disease follow‐up (%) (avg. 2009‐2016)	76.24 (75.55‐77.36)	82.42 (80.09‐83.95)	84.63 (82.98‐85.62)	87.42 (84.05‐87.86)

### 4.3 Classification accuracy

Sensitivity, specificity and positive predictive value for the primary case algorithm (3+ diagnosis codes plus 1+ medications), the alternative case algorithm (1+ diagnosis codes plus 1+ medication), and the non‐case algorithm (GI population) are shown in Table 3. Out of the 360 chart reviews of cases defined by the 3+ diagnosis codes plus 1+ medications algorithm, 14 were undetermined cases where the reviewers could not conclude the presence or absence of Crohn's disease among the patient. These patients were therefore excluded from the sensitivity and specificity analyses as they could not be categorized as a false positive or false negative. As expected, while the 1+ diagnosis plus 1+ medication algorithm delivered a higher sensitivity than the other case algorithm, whereas the 3+ diagnosis plus 1+ medications algorithm delivered a higher specificity and positive predictive value. Chart review (n = 40) suggested that the lower specificity in the 1+ diagnosis plus 1+ medications group was due primarily to either disease remission or to assigning a Crohn's diagnosis code to an encounter when in fact the patient was undergoing an initial evaluation that was subsequently found not to be Crohn's disease (ie, a “rule out Crohn's” visit). Positive predictive value improved by 10 percentage points by requiring at least three Crohn's diagnosis codes, with only a nine percentage point reduction in specificity.

**TABLE 3:** Sensitivity, specificity and positive predictive value of the Crohn's disease and non‐case algorithms selected to be compared to chart review

Algorithm	Sensitivity	Specificity	Positive predictive value
Case 3DM	0.91	0.95	0.94
Case 1DM	1	0.84	0.84
Non‐case (for Case 3DM)	0.95	0.91	0.92
Non‐case (for Case 1DM)	0.84	1	1

For discrepant cases identified using the 3+ diagnosis plus 1+ medication algorithm, two investigators conducted an error analysis to identify recurrent patterns. There was 100% agreement between the two investigators in the final disposition of the charts. There were two main reasons for these false positives (or type I errors): patients in whom an initial diagnosis of Crohn's disease was eventually reversed, and patients with indeterminate colitis or with suspected Crohn's that had not yet been biopsy confirmed. There were a small number of patients with other reasons for misclassification (n = 3) related to an initial concern for Crohn's that was eventually diagnosed as something else. No patients were identified where coding mistakes or “rule out” Crohn's disease resulted in the false positive result. Among those identified with the non‐case algorithm, the false negatives (or type II errors) included cases with insufficient follow‐up (eg, second opinions), and cases that represented early onset patients at the time of the data extract.

## 5 DISCUSSION

This study describes an overall process for developing computable phenotypes in PEDSnet, a large‐scale data network and Learning Health System, using Crohn's disease as a use case. Using a combination of informatics and clinical expertise and an internal validation and testing framework, we were able to confirm using records review that the process yielded a Crohn's disease computable phenotype with high sensitivity, specificity, and positive predictive value. We believe that this process can be generalized for other child health conditions within PEDSnet or other data sources that have similar data structures and definitions.

Compared to more manual approaches, automated computable phenotypes are highly advantageous for developing study cohorts in terms of efficiency and cost. This is especially important for diseases or health conditions that are rare, where a large population of patients would need to be screened to obtain reasonable sample sizes for studies. Given the high performance of the Crohn's disease computable phenotype developed in this project, we believe that PEDSnet, which now contains robust health care data on over six million children across eight children's hospitals, is a unique and valuable resource for this type of work more broadly. Similarly, collaboratives such as the Observational Health Data Sciences and Informatics (OHDSI)^15^ collaborative comprises a large number of health service datasets, structured in a common data model and terminology standards.

In this study, we evaluated the computable phenotype for Crohn's disease in a population of children and adolescents specifically. This is notable because for some diseases differences in disease impact and treatment patterns often require different phenotypes to track disease activity in children than in adults.^16^ However, the elements used on our Crohn's disease algorithms, including use of repeated presence of diagnoses to improve specificity, are similar to those used in adult populations to detect patients with Crohn's disease^17^ or ulcerative colitis.^18^ This suggests that our computable phenotype for Crohn's disease may perform well in both pediatric and adult populations, providing a consistent benchmark for studies comparing disease burden or treatment effectiveness across age groups, or to follow patients across the transition from pediatric to adult care. Additional work will be needed to assess whether cohorts ascertained at different ages by this approach share similar characteristics of disease subtype or activity.

While the sex and age distributions for Crohn's disease are consistent with a previous cross‐sectional study conducted on pediatric claims data for 2004 to 2009, the overall prevalence of Crohn's disease reported here is much higher.^13^ Reasons for this discrepancy are likely due to differences between the studies in how Crohn's disease was defined, the nature of underlying data, and the presence of tertiary care pediatric hospitals in PEDSnet, which likely have a higher prevalence of Crohn's compared to the general patient population. Additionally, the prior study was limited to commercially insured patients specifically, and case identification was based exclusively on claims data, rather than on EMR data.

For this study we developed a comprehensive SNOMED‐CT based diagnosis codeset, which allowed us to overcome discontinuities due to ICD version changes and low‐specificity codes, particularly in ICD‐9‐CM. We also utilized the multifaceted nature of EHR data, (eg, physician specialty, problem list entries, and medications) to refine the phenotype. Adding a medication criteria to the algorithm further enhanced the algorithm's accuracy. While not done in this study, a previous study in adult Crohn's patients demonstrated that adding the presence of specific narrative phrases associated with Crohn's disease from clinical notes (identified by natural language processing) improved the positive predictive value of the algorithm by 7% compared to an algorithm consisting of only ≥5 Crohn's ICD‐9 codes, and by 12% compared to an algorithm consisting of ≥1 outpatient Crohn's ICD‐9 code plus endoscopy.^19^ However, even with the “combined” algorithm that incorporated these phrases, the sensitivity was only 69% (specificity 97%), which is below that found in our study of 91%. This dichotomy suggests that the data included in PEDSNet may be somewhat more accurate that that used in the adult studies, which would not be unexpected given that PEDSNet maps data to the OMOP common data model, and performs rigorous and frequent data quality checks to ensure comparative validity of data across the different hospitals in the network.

An important consideration for this study is that for this project we chose to develop a Crohn's disease computable phenotype that optimized sensitivity along with specificity. For some projects other prioritizations may be preferred and more or less exclusive categorizations may be needed. For example, if the goal is to directly contact patients for potential enrollment in a Crohn's medication trial, one might want to prioritize specificity so as not to erroneously convey to patients and their families that they have Crohn's disease when in fact they do not (ie, apply a more exclusive algorithm like 3DM). On the other hand, if the goal is to screen children who have or are at risk for Crohn's for a longitudinal biomarker study, prioritizing sensitivity may be preferred (ie, a less exclusive algorithm like 1DM). Adding to this balance is the fact that for rare diseases like Crohn's, the pretest probability of having this disease is low, so false negatives are relatively rare. The approach described in this manuscript should be considered a general framework for computable phenotyping that can be altered depending on researchers' study needs.

This study should be considered in the context of several important limitations. First, while we describe an overall process for computable phenotype development, this particular study focused only on Crohn's disease. Further testing of the computable phenotype process as it is applied to other pediatric health conditions is needed to understand its generalizability for PEDSnet, as well as for other data sources. A second limitation is that, while one can reasonably calculate sensitivity and positive predictive values using the method and data source described, we reviewed only a subset of patient charts identified by our algorithms. It is nearly impossible to accurately define the frequency of false negatives in the entire population because of the possibility of incomplete data capture. A third limitation is that the use of broad fact ratios (here the presence of more Crohn's disease than ulcerative colitis diagnose), while producing simpler algorithms, may miss patients whose diagnoses converts from one to the other, whether through disease evolution or correction of miscoding.

## 6 CONCLUSIONS

We were able to successfully develop a computable phenotype for Crohn's disease from PEDSnet, a robust data source representing over six million children. Using a structured validation and evaluation process, we demonstrated that our EHR‐based algorithm for identifying Crohn's disease patients had a high sensitivity, specificity and positive predictive value. This computable phenotype will likely be of use for future studies to identify cohorts of pediatric Crohn's disease patients for retrospective observational analyses as well as prospective clinical trials. The overall approach we present can be applied to other pediatric disease states and conditions to advance child health research more broadly.

## CONFLICT OF INTEREST

Amanda Dempsey serves on Advisory Boards for Merck and Pfizer, and as a consultant to Pfizer for immunization‐related studies. She does not receive any research funding from these companies, and they played no role in this research. Michael Kappelman serves as a consultant to Johnson & Johnson, Abbvie, Pfizer, GlaxoSmithKline, and Lilly but these companies played no role in this research. All other authors have no conflicts of interest to declare.

## Supporting information


**Figure S1** Performance of Crohn's Disease Computable Phenotype Algorithms Compared to a Manually Curated list of Crohn's Disease Patients
**Table S1**. Association of Crohn's Disease CP Algorithm Components with Select GI Clinical Tests
**Table S2**. Crohn's disease codeset
**Table S3**. Ulcerative colitis codeset
**Table S4**. Indeterminate colitis codeset

## APPENDIX 1

### PEDSnet Computable Phenotype Working Group Authors

Jennifer L. Dotson, MD, MPH

Division of Gastroenterology, Hepatology and Nutrition, The Ohio State University College of Medicine, Columbus, OH, USA

Center for Innovation in Pediatric Practice, The Research Institute at Nationwide Childrens Hospital, Columbus, OH, USA

Maura A. Downing, MHA

Division of Gastroenterology, Hepatology and Nutrition, Department of Pediatrics, Childrens Hospital of Philadelphia, Philadelphia, PA, USA

Katherine D. Hawley, BSN, RN

Center for Child Health, Behavior, and Development, Seattle Childrens Research Institute, Seattle, WA, USA

Lauren Kluge, MS

James M. Anderson Center for Health Systems Excellence, Cincinnati Childrens Hospital Medical Center, Cincinnati, OH, USA

Timothy M. Maul, CCP, PhD

Division of Cardiac Surgery, Nemours Childrens Hospital, Orlando, FL, USA

Department of Bioengineering

University of Pittsburgh, Pittsburgh, PA, USA

Matthew W. Miller, MS

Department of Biomedical and Health Informatics

Childrens Hospital of Philadelphia, Philadelphia, PA, USA

Lise E. Nigrovic, MD, MPH

Division of Emergency Medicine

Boston Childrens Hospital, Boston, MA, USA
