# Supplementary material for: Development and evaluation of an EHR‐based computable phenotype for identification of pediatric Crohn's disease patients in a National Pediatric Learning Health System
Source: Learn Health Syst. 2020 Aug 28;4(4):e10243. doi: 10.1002/lrh2.10243 (PMC7556434; doi:10.1002/lrh2.10243)
Supplement: Supplementary file 1 — Figure S1 Performance of Crohn's Disease Computable Phenotype Algorithms Compared to a Manually Curated list of Crohn's Disease Patients Table S1. Association of Crohn's Disease CP Algorithm Components with Select GI Clinical Tests Table S2. Crohn's disease codeset Table S3. Ulcerative colitis codeset Table S4. Indeterminate colitis codeset [file LRH2-4-e10243-s001.docx]

**Supplemental Material (Online Only) for:**

**Development and Evaluation of an EHR-based Computable Phenotype for Pediatric Crohn’s Disease**

**Figure S1. Performance of Crohn’s Disease Computable Phenotype Algorithms Compared to a Manually Curated list of Crohn’s Disease Patients**

Figure S1 shows the results of comparison of the phenotype algorithms results with variable diagnoses and the presence or absence of Crohn’s medications with a manually curated registry of Crohn’s disease patients at a single PEDSnet institution. (“d”= number of diagnosis, “m”= evidence of being prescribed one or more medications commonly used to treat Crohn’s disease).

**Table S1. Association of Crohn’s Disease CP Algorithm Components with Select GI Clinical Tests**

| Outcome | Crohn’s Disease Diagnosis Encounter  (1, 2, 3, 4, 5+) | Crohn’s Disease Medications  (yes/no) |
| --- | --- | --- |
| Endoscopy | 1.47  (1.45-1.49) | 3.57  (3.47-3.68) |
| GI Radiographic Exam | 1.67  (1.65-1.69) | 4.87  (4.7-5.04) |

**Table S2. Crohn’s disease codeset**

| SNOMED | ICD9 | ICD10 |
| --- | --- | --- |
| \| \| 7620006 \| \| --- \| \| 34000006 \| \| 71833008 \| \| 56689002 \| \| 196977009 \| \| 196578009 \| \| 201805000 \| \| 201728006 \| \| 234999001 \| \| 413276006 \| \| 50440006 \| \| 414153008 \| \| 414154002 \| \| 56287005 \| \| 397173003 \| \| 91390005 \| \| 3815005 \| \| 410485009 \| \| 38106008 \| \| 397172008 \| \| 399946006 \| \| 402377001 \| \| 235607002 \| \| 61424003 \| \| 402376005 \| \| 402375009 \| \| 402378006 \| \| 70622003 \| \| 235664007 \| \| 235796008 \| \| 1085751000119100 \| \| 1085761000119100 \| \| 1085771000119100 \| \| 1085781000119100 \| \| 1085791000119100 \| \| 1085801000119100 \| \| 1085811000119100 \| \| 1085821000119100 \| \| 1085831000119100 \| \| 1085841000119100 \| \| 1085851000119100 \| \| 1085861000119100 \| \| 1085871000119100 \| \| 1085881000119100 \| \| 1085891000119100 \| \| 1085901000119100 \| \| 1085911000119100 \| \| 1085931000119100 \| \| 1085941000119100 \| \| \| --- \| --- \| --- \| --- \| --- \| --- \| --- \| --- \| --- \| --- \| --- \| --- \| --- \| --- \| --- \| --- \| --- \| --- \| --- \| --- \| --- \| --- \| --- \| --- \| --- \| --- \| --- \| --- \| --- \| --- \| --- \| --- \| --- \| --- \| --- \| --- \| --- \| --- \| --- \| --- \| --- \| --- \| --- \| --- \| --- \| --- \| --- \| --- \| --- \| --- \| \|  \| \|  \| \|  \| \|  \| \|  \| \|  \| \|  \| \|  \| \|  \| \|  \| \|  \| \|  \| \|  \| \|  \| \|  \| \|  \| \|  \| \|  \| \|  \| \|  \| \|  \| \|  \| \|  \| \|  \| \|  \| \|  \| \|  \| \|  \| \|  \| \|  \| \|  \| \|  \| \|  \| \|  \| \|  \| \|  \| \|  \| \|  \| \|  \| \|  \| \|  \| \|  \| \|  \| \|  \| \|  \| \|  \| \|  \| \|  \| | \| 555.1 \| \| --- \| \| 555.9 \| \| 555 \| \| 555.2 \| | \| K50.1 \| \| --- \| \| K50.111 \| \| K57.50 \| \| K50.114 \| \| K50.10 \| \| K50.911 \| \| K50 \| \| K57.30 \| \| K50.9 \| \| K50.914 \| \| K50.913 \| \| K50.90 \| \| K57.33 \| \| K50.80 \| \| K50.814 \| \| K57.41 \| \| K50.8 \| \| K50.812 \| \| K50.811 \| \| K50.813 \| \| K50.014 \| \| K50.011 \| \| K57.51 \| \| K50.0 \| \| K50.00 \| \| K57.52 \| \|  \| \| K50.119 \| \| K50.118 \| \| K50.11 \| \| K50.113 \| \| K50.112 \| \| K50.819 \| \| K50.818 \| \| K50.81 \| \| K57.40 \| \| K57.53 \| \| K50.019 \| \| K50.018 \| \| K50.01 \| \| K50.013 \| \| K50.012 \| \| K50.919 \| \| K50.91 \| \| K50.918 \| \| K50.912 \| \| K57.31 \| \| K57.32 \| |

| **SNOMED code** | **ICD9 code** | **ICD10 code** |
| --- | --- | --- |
| 7620006 | 555.1 | K50.1 |
| 7620006 | 555.1 | K50.111 |
| 7620006 | 555.1 | K57.50 |
| 7620006 | 555.1 | K50.114 |
| 7620006 | 555.1 | K50.10 |
| 34000006 | 555.9 | K50.911 |
| 34000006 | 555 | K50.911 |
| 34000006 | 555.9 | K50 |
| 34000006 | 555 | K50 |
| 34000006 | 555.9 | K57.30 |
| 34000006 | 555 | K57.30 |
| 34000006 | 555.9 | K50.9 |
| 34000006 | 555 | K50.9 |
| 34000006 | 555.9 | K50.914 |
| 34000006 | 555 | K50.914 |
| 34000006 | 555.9 | K50.913 |
| 34000006 | 555 | K50.913 |
| 34000006 | 555.9 | K50.90 |
| 34000006 | 555 | K50.90 |
| 34000006 | 555.9 | K57.33 |
| 34000006 | 555 | K57.33 |
| 71833008 | 555.2 | K50.80 |
| 71833008 | 555.2 | K50.814 |
| 71833008 | 555.2 | K57.41 |
| 71833008 | 555.2 | K50.8 |
| 71833008 | 555.2 | K50.812 |
| 71833008 | 555.2 | K50.811 |
| 71833008 | 555.2 | K50.813 |
| 56689002 | 555 | K50.014 |
| 56689002 | 555 | K50.011 |
| 56689002 | 555 | K57.51 |
| 56689002 | 555 | K50.0 |
| 56689002 | 555 | K50.00 |
| 56689002 | 555 | K57.52 |
| 1085761000119100 |  | K50.119 |
| 1085761000119100 |  | K50.118 |
| 1085761000119100 |  | K50.10 |
| 1085761000119100 |  | K50.11 |
| 1085771000119100 |  | K50.113 |
| 1085781000119100 |  | K50.112 |
| 1085811000119100 |  | K50.819 |
| 1085811000119100 |  | K50.818 |
| 1085811000119100 |  | K50.81 |
| 1085841000119100 |  | K57.40 |
| 1085851000119100 |  | K57.53 |
| 1085861000119100 |  | K50.019 |
| 1085861000119100 |  | K50.018 |
| 1085861000119100 |  | K50.01 |
| 1085871000119100 |  | K50.013 |
| 1085881000119100 |  | K50.012 |
| 1085911000119100 |  | K50.919 |
| 1085911000119100 |  | K50.91 |
| 1085911000119100 |  | K50.90 |
| 1085911000119100 |  | K50.918 |
| 1085931000119100 |  | K50.912 |
| 1085931000119100 |  | K57.31 |
| 1085941000119100 |  | K57.32 |

**Table S3. Ulcerative colitis codeset**

| **SNOMED Codes** | **ICD9 Codes** | **ICD10 codes** |
| --- | --- | --- |
| \| 444546002 \| \| --- \| \| 444548001 \| \| 445243001 \| \| 441971007 \| \| 442159003 \| \| 64766004 \| \| 52506002 \| \| 13470001 \| \| 12109003 \| \| 128600008 \| \| 14311001 \| \| 24829000 \| \| 201727001 \| \| 201807008 \| \| 404908004 \| \| 410484008 \| \| 414156000 \| \| 78324009 \| \| 78712000 \| \| 1092841000119100 \| \| 1092861000119100 \| \| 1085161000119100 \| \| 1085261000119100 \| \| 1092871000119100 \| \| 1092851000119100 \| \| 1092881000119100 \| \| 1085131000119100 \| \| 1085141000119100 \| \| 1085151000119100 \| \| 1085171000119100 \| \| 1085231000119100 \| \| 1085241000119100 \| \| 1085251000119100 \| \| 1085271000119100 \| | \| 556.6 \| \| --- \| \| 556.9 \| \| 556.5 \| \| 556 \| \| 556.8 \| \| 556.3 \| \| 556.1 \| | \| K57.9 \| \| --- \| \| K57.11 \| \| K57.13 \| \| K51.5 \| \| K57.12 \| \| K51.50 \| \| K57.10 \| \| K51.51 \| \| K57.8 \| \| K51.0 \| \| K51.011 \| \| K51.014 \| \| K51.013 \| \| K51 \| \| K57.30 \| \| K51.90 \| \| K51.00 \| \| K57.21 \| \| K51.9 \| \| K51.80 \| \| K51.8 \| \| K51.914 \| \| K51.313 \| \| K51.311 \| \| K51.3 \| \| K51.30 \| \| K57.2 \| \| K51.314 \| \| K57.3 \| \| K51.514 \| \| K51.814 \| \| K57.20 \| \| K51.813 \| \| K51.513 \| \| K51.913 \| \| K51.012 \| \| K51.312 \| \| K51.912 \| \| K51.512 \| \| K51.812 \| \| K51.518 \| \| K51.91 \| \| K51.918 \| \| K51.81 \| \| K51.818 \| \| K51.519 \| \| K51.919 \| \| K51.819 \| \| K51.811 \| \| K51.911 \| \| K51.511 \| \| K51.019 \| \| K51.01 \| \| K51.018 \| \| K57.4 \| \| K51.318 \| \| K51.319 \| \| K51.31 \| \| K57.5 \| |

| **SNOMED Code** | **ICD9 Code** | **ICD10 Code** |
| --- | --- | --- |
| 445243001 |  | K57.9 |
| 445243001 |  | K57.11 |
| 445243001 |  | K57.13 |
| 445243001 |  | K51.5 |
| 445243001 |  | K57.12 |
| 445243001 |  | K51.50 |
| 445243001 |  | K57.10 |
| 445243001 |  | K51.51 |
| 445243001 |  | K57.8 |
| 442159003 | 556.6 | K51.0 |
| 442159003 | 556.6 | K51.011 |
| 442159003 | 556.6 | K51.014 |
| 442159003 | 556.6 | K51.013 |
| 64766004 | 556.9 |  |
| 64766004 | 556.5 | K51 |
| 64766004 | 556 | K51 |
| 64766004 | 556.8 | K51 |
| 64766004 | 556.9 |  |
| 64766004 | 556.5 | K57.30 |
| 64766004 | 556 | K57.30 |
| 64766004 | 556.8 | K57.30 |
| 64766004 | 556.9 |  |
| 64766004 | 556.5 | K51.90 |
| 64766004 | 556 | K51.90 |
| 64766004 | 556.8 | K51.90 |
| 64766004 | 556.9 |  |
| 64766004 | 556.5 | K51.00 |
| 64766004 | 556 | K51.00 |
| 64766004 | 556.8 | K51.00 |
| 64766004 | 556.9 |  |
| 64766004 | 556.5 | K57.21 |
| 64766004 | 556 | K57.21 |
| 64766004 | 556.8 | K57.21 |
| 64766004 | 556.9 |  |
| 64766004 | 556.5 | K51.9 |
| 64766004 | 556 | K51.9 |
| 64766004 | 556.8 | K51.9 |
| 64766004 | 556.9 |  |
| 64766004 | 556.5 | K51.80 |
| 64766004 | 556 | K51.80 |
| 64766004 | 556.8 | K51.80 |
| 64766004 | 556.9 |  |
| 64766004 | 556.5 | K51.8 |
| 64766004 | 556 | K51.8 |
| 64766004 | 556.8 | K51.8 |
| 64766004 | 556.9 |  |
| 64766004 | 556.5 | K51.914 |
| 64766004 | 556 | K51.914 |
| 64766004 | 556.8 | K51.914 |
| 52506002 | 556.3 | K57.8 |
| 52506002 | 556.3 | K51.313 |
| 52506002 | 556.3 | K51.311 |
| 52506002 | 556.3 | K51.3 |
| 52506002 | 556.3 | K57.9 |
| 52506002 | 556.3 | K51.30 |
| 52506002 | 556.3 | K57.2 |
| 52506002 | 556.3 | K51.314 |
| 52506002 | 556.3 | K57.3 |
| 13470001 | 556.1 |  |
| 1092841000119100 |  | K51.514 |
| 1092841000119100 |  | K51.914 |
| 1092841000119100 |  | K51.814 |
| 1092841000119100 |  | K57.20 |
| 1092861000119100 |  | K51.813 |
| 1092861000119100 |  | K51.513 |
| 1092861000119100 |  | K51.913 |
| 1085161000119100 |  | K51.012 |
| 1085261000119100 |  | K51.312 |
| 1092871000119100 |  | K51.912 |
| 1092871000119100 |  | K51.512 |
| 1092871000119100 |  | K51.812 |
| 1092851000119100 |  | K51.518 |
| 1092851000119100 |  | K51.91 |
| 1092851000119100 |  | K51.918 |
| 1092851000119100 |  | K51.81 |
| 1092851000119100 |  | K51.818 |
| 1092851000119100 |  | K51.519 |
| 1092851000119100 |  | K51.919 |
| 1092851000119100 |  | K51.819 |
| 1092881000119100 |  | K51.811 |
| 1092881000119100 |  | K51.911 |
| 1092881000119100 |  | K51.511 |
| 1085131000119100 |  | K57.20 |
| 1085141000119100 |  | K51.019 |
| 1085141000119100 |  | K51.01 |
| 1085141000119100 |  | K51.018 |
| 1085151000119100 |  | K57.21 |
| 1085231000119100 |  | K57.4 |
| 1085241000119100 |  | K51.318 |
| 1085241000119100 |  | K51.319 |
| 1085241000119100 |  | K51.31 |
| 1085251000119100 |  | K57.5 |

**Table S4. Indeterminate colitis codeset**

| **SNOMED Code** |
| --- |
| 235746007 |
